# Evaluating the efficacy of three classical EEG paradigms in the discrimination of bipolar depression

**DOI:** 10.3389/fpsyt.2025.1545132

**Published:** 2025-05-29

**Authors:** Chen Yang, Yao Pi, Weijie Wang, Ying Huang, Nan Tang, Hong Wang, Shenglin Wen

**Affiliations:** ^1^ Department of Psychology, The Fifth Affiliated Hospital, Sun Yat-sen University, Zhuhai, China; ^2^ The School of Biomedical Engineering, Sun Yat-Sen University, Shenzhen, China; ^3^ Department of Child Psychiatry and Rehabilitation, Shenzhen Maternity and Child Healthcare Hospital, Southern Medical University, Shenzhen, Guangdong, China

**Keywords:** bipolar depression (BD), electroencephalogram (EEG), experimental paradigms, phase lag index (PLI), neuropsychological assessments, classification

## Abstract

**Objective:**

Given the lack of consensus regarding the optimal EEG paradigm for identifying bipolar depression (BD), this study sought to systematically evaluate the efficacy of three classic EEG paradigms—eyes open, eyes closed, and free viewing—in diagnosing BD.

**Methods:**

EEGs were collected from 28 individuals diagnosed with BD and 42 healthy controls(HCs) across three experimental conditions: eyes closed, eyes open, and free viewing. Sociodemographic data and neuropsychological testing were also collected. This research investigated notable variations in brain functional connectivity between the two groups across paradigms, the correlation of features with neuropsychological assessments, and classification outcomes.

**Results:**

The results demonstrated that under the eyes-closed paradigm, significant differences in the Phase Lag Index (PLI) were consistently observed across the δ, θ, β, and γ frequency bands. This paradigm also featured the highest number of electrodes significantly correlated with cognitive scales. Furthermore, the eyes-closed condition achieved the highest accuracy in bipolar depression recognition, with the Random Forest classifier yielding the highest accuracy of 79.43% and an F1 score of 76.82%. These findings underscore the eyes closed paradigm as a superior, straightforward EEG experimental approach for the diagnosis of bipolar depression.

**Conclusions:**

This study indicates that the eyes closed experimental paradigm more effectively demonstrates the electrophysiological disparities between patients with BD and HCs, in comparison to the eyes open paradigm and the action observation-based free viewing paradigms, as determined through the analysis of various outcome metrics.

## 1 Introduction

Bipolar disorder is a severe mood disorder, primarily characterized by extreme fluctuations in mood states, alternating episodes of depression and (hypo)mania (1). The initial onset of bipolar disorder is typically depressive, with depressive episodes lasting significantly longer throughout the course of the illness than manic or hypomanic episodes. The severity of depressive symptoms surpassing mania typically manifests during the developmental stages of children and adolescents, imparting adverse effects on education and vocational prospects (2). Early onset is associated with a diminished prognosis. Furthermore, acute episodes precipitate profound cognitive and psychosocial functional impairments, significantly disrupting attention, cognitive flexibility, executive function, and working memory (3, 4). The prevalence of bipolar disorder has been rising with the evolution of modern society, along with its high disability rate and increased risk of suicide (5), thereby exerting a significant social burden (6). Individuals with bipolar disorder frequently receive incorrect diagnoses, are overdiagnosed, or are only diagnosed several years after disease onset, leading to a worse disease prognosis (3, 7). Therefore, early identification of bipolar disorders is crucial.

Recent investigations have increasingly concentrated on the neural biomarkers of bipolar disorder, which have the potential for precise and timely diagnosis (8). Among the various non-invasive monitoring methods, electroencephalographic (EEG) captures macroscopic temporal dynamics of brain activity by measuring scalp electrical potentials, thereby being considered a suitable tool (9). EEG-derived datasets are increasingly utilized in bipolar disorder research (7). Studies on biomarkers of bipolar disorder have primarily focused on aspects such as the P300 wave, functional connectivity, and oscillation frequency bands, with a growing interest in the application of machine-learning techniques (10–13). Despite adopting various experimental paradigms over the past decade, including resting-state and task-related measurements during emotional or cognitive tasks (14). There is no consensus on the optimal experimental paradigm for exploring EEG biomarkers in bipolar disorder and healthy controls (HCs) (9, 15). Current mainstream experimental paradigms include eyes closed, eyes open, and free viewing protocols, with the latter encompassing the free viewing of images and videos, as well as cognitive tasks (16–18). This study employed a video-based free viewing paradigm.

Research using the eyes closed paradigm revealed decreased alpha (α) power and increased delta (δ) and theta (θ) activity in the EEG profiles of initial bipolar disorder episodes relative to HCs (19). Some studies have found that bipolar disorder exhibits higher power across all frequencies, including the beta (β) and gamma (γ) bands than HCs. Patients with bipolar disorder show greater coherence in the α band within the parietal-temporal and central parietal regions, while interhemispheric coherence in the δ band of the frontal regions is lower (20). Similarly, another study found higher coherence in the frontal and occipital cortex, particularly in the frontal regions, compared to HCs (21). Studies employing an eyes open experimental paradigm have observed disruptions in frontal lobe slow-wave oscillations, with increased activity in the δ, θ, and α bands (22). Research has also found increased α-wave power in the central-frontal and right parietal regions in bipolar disorder (23). However, another study found slowed α-wave activity during depressive episodes in adolescents with bipolar disorder, which has been associated with decreased cognitive function (24). Within free viewing paradigms, the saccadic eye movement approach suggests γ coherence as a potential marker for cognitive dysfunction in manic phases (25). Furthermore, EEG responses to emotional facial expressions have been instrumental in distinguishing unipolar from bipolar depression (BD), highlighting the diagnostic utility of EEG activity (15).

This study explored the contributions of three mainstream experimental paradigms to electroencephalographic biomarkers of bipolar disorders. We employed video-based observations to investigate the free viewing paradigm. This approach is predicated on the premise that central brain regions exhibit a sensorimotor α-rhythm during eyes open states or the observation and execution of actions. This rhythm has a frequency range of 8–13 Hz and is a variant of the α-rhythm (26, 27). Numerous studies suggest that this sensorimotor α-rhythm may serve as an indicator that may aid in the discovery of the pathophysiological mechanisms of bipolar disorder (28; S. C. 29). A study on patients with bipolar disorder found that the bipolar disorder group exhibited lower levels of sensorimotor α-rhythm suppression compared to HCs, possibly indicating social cognitive difficulties. Kim et al. (28) examined the neural activity of euthymic bipolar disorder patients in a new virtual reality social cognitive task. They found that bipolar disorder participants exhibited slower reaction times to the emotional component of the task (despite comparable accuracy), particularly in the inferior frontal cortex, pre-motor cortex, and insula. Based on this, we chose a free viewing paradigm for video observation, with participants watching videos of goal-directed actions. Research suggests that observing videos of hand movements can enhance brain activity patterns (30). We selected videos from a dynamic action stimuli database (31) and clipped actions familiar to Chinese participants to create the free viewing videos used in our study.

Numerous studies have identified considerable variations in the results of biomarkers for bipolar disorder, which may be attributed to applying different experimental paradigms (4, 9, 32, 33). The extant data are insufficient for deducing trends or computing consistency scores and are often poorly validated (34). Consequently, this study has selected three distinct paradigms, conducting a comprehensive analysis of various electroencephalographic signals to investigate the differential diagnostic capabilities of each paradigm.

## 2 Methods and materials

Before starting the study, ethical approval was obtained from the Ethics Committee of the Fifth Affiliated Hospital of Sun Yat-sen University.

### 2.1 Participants

Participants with bipolar disorder were recruited from the psychiatric inpatient service and diagnosed using DSM-5 criteria by two independent and experienced psychiatrists, one of whom was the patient’s treating psychiatrist. All participants in the patient group were assessed to be in a depressive state at the time of evaluation, with none meeting the criteria for a current manic, hypomanic, or mixed episode. All patients with bipolar disorder were diagnosed with a bipolar depressive episode. The HC group was recruited using oral advocacy and promotional posters. Both groups underwent semi-structured interviews to assess their medical history. All participants completed a demographic questionnaire, trail making test (TMT), digit span test (DST), and symbol digit modalities test (SDMT). Additionally, symptoms of depression and mania were assessed using the Hamilton depression scale-24 items (HAMD-24; 35)and the young mania rating scale (YMRS) (36).

Exclusion criteria included reported comorbid intellectual disability and other psychiatric disorders, recent electroconvulsive therapy in the past six months, recent substance abuse or dependence in the past six months, and severe physical or organic diseases, such as cardiovascular or major orthopedic diseases, severe developmental disorders, and neurological diseases. This study included 70 participants; 28 were diagnosed with BD, and 42 were HCs. The demographic and clinical data of the participants are summarized in **Table 1**.

**Table 1:** Demographic and clinical characteristics.

Parameters	BD,n=28	HCs,n=42	T(*X* ^2^)	*p*
Age (years)	21.11 ± 4.42	22.86 ± 2.09	-1.95	.06^a^
Gender (female, male)	20,8	25,17	*X* ^2^ = 1.03	.31^b^
Years of education	12.82 ± 1.89	13.24 ± 1.53	.64	.31^a^
Duration of illness in years	4.19 ± 3.66	–	–	–
YMRS score	1.25 ± 1.04	.50 ± .67	3.37	.00^a^
HAMD-24 score	34.29 ± 9.29	2.29 ± 1.66	18.03	.00^a^
TMT-A(s)	27.45 ± 7.09	26.27 ± 8.52	.51	.54^a^
TMT-B(s)	43.25 ± 8.56	32.64 ± 7.90	.45	.00^a^
DST score	11.75 ± 1.29	15.09 ± 2.38	.00	.00^a^
SDMT score	63.75 ± 11.45	73.17 ± 12.51	.57	.00 ^a^

Values in the tables are presented by default as counts, mean ± standard deviation, unless otherwise indicated.

YRMS, Young Mania Rating Scale; HAMD-24, Hamilton Depression Scale-24 (HAMD-24); TMT, Trail Making Test; DST, Digit Span Test; SDMT, Symbol Digit Modalities Test.

BD, individuals with bipolar depression; HCs, healthy controls.

^a^ T-tests; ^b^ Chi-square test.

### 2.2 Experimental design and procedure

#### 2.2.1 Neuropsychological testing

On the same morning of EEG acquisition, neurocognitive testing was conducted. These tests were completed by 28 BD patients and 42 HCs. A battery of neuropsychological tests was used to assess the following cognitive domains: (a) The Trail Making Test (TMT) is a neuropsychological test used to assess executive functions (EFs) (37). The test consists of two parts (A and B). TMT-A is typically considered a measure of visual search and processing speed, where participants must sequentially connect the numbers 1 to 25 as quickly as possible. The score is the time taken to complete the task (in seconds), with shorter times indicating better performance (38) (39). TMT-B is considered to more broadly assess psychological flexibility and executive functions (40–42). (b) The digit span test (DST) evaluates working memory and cognitive flexibility. It includes forward and backward tasks. The forward digit span test comprises a series of digits ranging from 2 to 12, which the examiner recites at a rate of one digit per second, and participants must repeat in the same order. The backward digit span test comprises digits ranging from 2 to 10, recited forward by the examiner, and participants must recite them backward. The score is the total sum of the last level of digits passed (43). (c) The symbol digit modalities test (SDMT) is an advanced form of the digit-symbol test that assesses attention, visual scanning, and motor speed. Individuals must identify nine symbols corresponding to the digits 1 to 9 and practice writing the correct number beneath each symbol. Then, they manually fill the blanks with the corresponding number under each symbol. Participants are given 90 s to complete the written test. The score was calculated by totaling the correct answers for each section (44).

#### 2.2.2 EEG acquisition and electrophysiological recording

During EEG acquisition, participants sat quietly in a comfortable armchair and sequentially completed the following three conditions: 1) eyes closed, 2) eyes open, and 3) free viewing. eyes closed Participants sat in the chair for 3 min in the eyes closed condition. The instruction was, ‘Please close your eyes, relax, and do not think about anything.’ In the eyes open condition, participants sat in the chair for 3 min with the instruction, ‘Please open your eyes, relax, try to minimize blinking, and do not think about anything’. Under the free viewing condition, participants sat in the chair for 3 min and 20s with the instruction ‘Please view every action in the video, but do not make any behavioral movements’. The duration of the free viewing videos was 3 min and 20 s.

The EEG signals were recorded using a 32-channel system (Nicolet Monitor, USA) with an electrode cap, and recording was performed according to the 10–20 international system. The reference electrodes were placed midway between CPz and AFz. The recorded signals were digitized at a sampling frequency of 250 Hz, with impedance for all electrodes less than 10 kohm. EEG data were preprocessed and analyzed using MATLAB (R2022a; Mathworks, Natick, MA, USA) and EEGLAB v14.0. Initially, the EEG signals were re-referenced using average reference montage. Subsequently, bandpass filtering from 1 to 49 Hz and notch filtering at 50 Hz were applied to remove high-frequency and power-line noise contamination. Independent component analysis (ICA) was used to remove eye movement and blink artifacts. Next, EEG signals from each channel were segmented into non-overlapping epochs of 6 s. Power spectral density (PSD) and differential entropy (DE) features were computed for all segments across different frequency bands. Additionally, a Butterworth IIR bandpass filter was applied separately to data from all channels to obtain δ (1–4 Hz) and β (12–30 Hz) waves (zero-phase shift). Subsequently, the Hilbert transformation was used to obtain phase and amplitude information for each channel, from which debiased phase-amplitude cross-frequency coupling (dPAC) and amplitude-amplitude coupling (AAC) were calculated.

#### 2.2.3 Phase Lag Index Functional Connectivity

The phase lag index (PLI), a measure of phase synchronization, was used to quantify the functional connectivity between each pair of EEG channels (45, 46):


$$PLI_{\left(f,t\right)}=\left|\frac{1}{M}\sum_{m=1}^{M}\text{sgn}\left(△\varphi_{a,b}^{m}\left(f,t\right)\right)\right|$$

where $\;△\varphi_{a,b}^{m}\left(f,t\right)$ stands for the phase difference between channel a and b at frequency f and time t of trial m, M stands for the number of trials, and sgn indicates the sign (–1 for negative values, +1 for positive values, and 0 for zero values).

In this study, PLI values were calculated for three experimental paradigms using the aforementioned equation. PLI values were averaged across δ (1–4 Hz), θ (4–8 Hz), α (8–12 Hz), β (12–30 Hz), and γ (> 30 Hz) frequency bands (47) and within a time window of 0.1 to 11 s to derive a weighted 19×19 functional connectivity matrix, serving as a candidate feature for each participant. PLI was calculated for 19 electrodes representing brain regions (FP1, FP2, F3, F4, F7, F8, FZ, T7, T8, P7, P8, C3, C4, CZ, P3, P4, PZ, O1, and O2). The PLI represents the connection strength between electrode pairs, ranging between 0 and 1, with higher values indicating stronger nonzero phase locking (48).

#### 2.2.4 Features and correlations

In this study, EEG signals were decomposed into functionally distinct frequency bands (δ, θ, α, β, and γ). PSD for each band, DE for each band, as well as δ dPAC and AAC of δ and β bands, were computed. Twelve features were extracted in this study. To ascertain the relationship between EEG signal and cognitive function, Spearman correlation analysis was conducted to examine the correlations between TMT-A, TMT-B, DST, and SDMT scores and 12 significant features under the three experimental paradigm conditions. Correlations were deemed significant at p< 0.05.

#### 2.2.5 Classification

Based on these features, we employed six classifiers, including ensemble, K-nearest neighbours (KNN), Naive Bayes, Random Forest, support vector machine (SVM), and decision tree (DT), to evaluate the BD identification performance under the eyes closed, eyes open, and free viewing experimental paradigms. The effectiveness of BD identification was measured using accuracy and F1 score. In this study, the selected classifiers are classical machine learning classifiers, extensively applied in depression recognition research (49).

- Ensemble, an ensemble learning algorithm, uses AdaBoost M1 to train weak classifiers (usually decision tree stumps or single-layer decision trees). This method combines their results by weighting them to construct a more robust classifier. The basic learner parameters were set to decision trees, with the number of basic learners fixed at 100 (50, 51).
- K-nearest neighbours(KNN) computed the distance between a test sample and all training samples, typically using the Euclidean distance metric. The prediction of the test sample’s category was determined by a majority vote among the K closest training samples (52). The parameter K of KNN was fixed constant 5 (53).
- Naive Baye is a simple yet effective classifier based on Bayes’ theorem. It calculated the class conditional and prior probabilities, deriving the posterior probabilities. The class with the highest posterior probability was selected as the prediction (54).
- Random Forest mitigates the risk of overfitting by integrating multiple decision trees. It performs well with high-dimensional and large datasets, requiring minimal parameter tuning (55).
- The SVM was introduced by (56). Its basic idea is to classify samples by finding the hyperplane with the largest distance between samples. The regularization parameter C of the Linear-SVM was empirically set to 0.25 (57).
- DT is a supervised learning classifier based on a tree structure. They construct recursive decision rules from training samples and predict the categories of test samples according to these trained rules (58).

### 2.3 Statistical analysis

Statistical analysis was performed using SPSS (v25.0) and MATLAB. Continuous variables were presented as mean ± standard deviation, and categorical variables were denoted as numbers. T-tests and Pearson’s chi-square tests were utilized to compare the baseline characteristics between the two groups. The connectivity strength between the two groups was compared using nonparametric Wilcoxon signed-rank tests with False Discovery Rate (FDR) correction to control for multiple comparison issues. Uncorrected p-values as ‘p,’ and FDR corrected p-values were presented as ‘p_FDR_.’ The independent samples t-test was employed to assess the significance of differences in features between BD and HC groups (utilizing the MATLAB function ttest2). The MATLAB ttest2 function facilitated this comparison, allowing for a robust assessment of the significance level. Additionally, correlation analysis was conducted to investigate the relationship between EEG features and cognitive assessment scores, with Spearman’s correlation analysis (implemented using the MATLAB function corr), providing a reliable measure of the strength and direction of the relationship between the variables of interest. p< 0.05 was considered a statistically significant difference.

## 3 Results

### 3.1 Demographic characteristics

**Table 1** depicts the demographic and clinical characteristics of the study sample. There were no statistically significant differences between the BD group and the HCs regarding age, gender, or years of education. The YMRS scores of BD and HC groups were< 5 points. The TMT-A scores did not differ significantly between the BD group and the HCs. However, TMT-B, DST, and SDMT scores differed statistically significantly.

### 3.2 Brain functional connectivity

**Figure 1:**
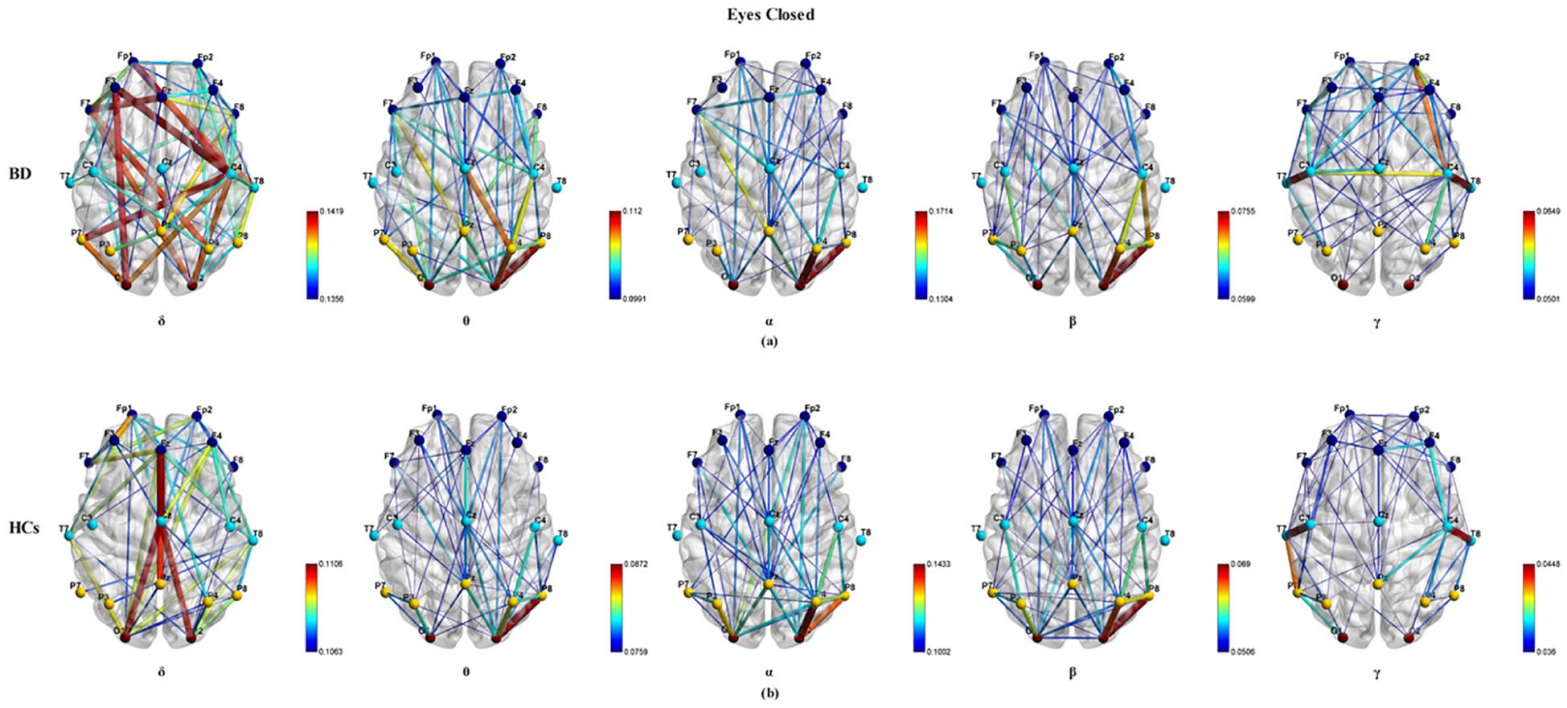
The visualization of the group average PLI functional connectivity for BD **(a)** and HCs **(b)** under the eyes closed experimental paradigm across five frequency bands (δ, θ, α, β, and γ), with a sparsity of 0.3.

PLI was computed for all electrode pairs in the δ, θ, α, β, and γ bands under the three experimental paradigms. **Figures 1a, b** illustrate the group averaged PLI functional connectivity patterns in the δ, θ, α, β, and γ frequency bands for BD patients and HCs under the eyes closed experimental paradigm condition. We observed significant differences in the functional connectivity between the two groups across all electrode pairs in the δ, θ, β, and γ frequency bands (P_FDR<_ 0.05), the BD group demonstrating increased functional connectivity compared to HCs. A qualitative observation in the δ frequency band revealed that BD patients exhibited stronger long-range connections between the frontal and occipital cortical regions compared to HCs (P_FDR<_ 0.05). In the θ frequency band, BD patients showed more short-range connections between the parietal and occipital cortical regions and fewer long-range connections between the frontal and the parietal/occipital cortical regions compared to HCs (P_FDR<_ 0.05). In the β frequency band, there were fewer long-range connections between the frontal and parietal/occipital cortical regions in BD patients relative to HCs (P_FDR<_ 0.05). In the γ frequency band, BD patients displayed stronger long-range connections between the frontal cortical regions and the visual cortex, and short-range connections across hemispheres than HCs (P_FDR<_ 0.05). No significant effects of functional connectivity between the two groups were observed in the α frequency band (P_FDR_ > 0.05).

**Figure 2:**
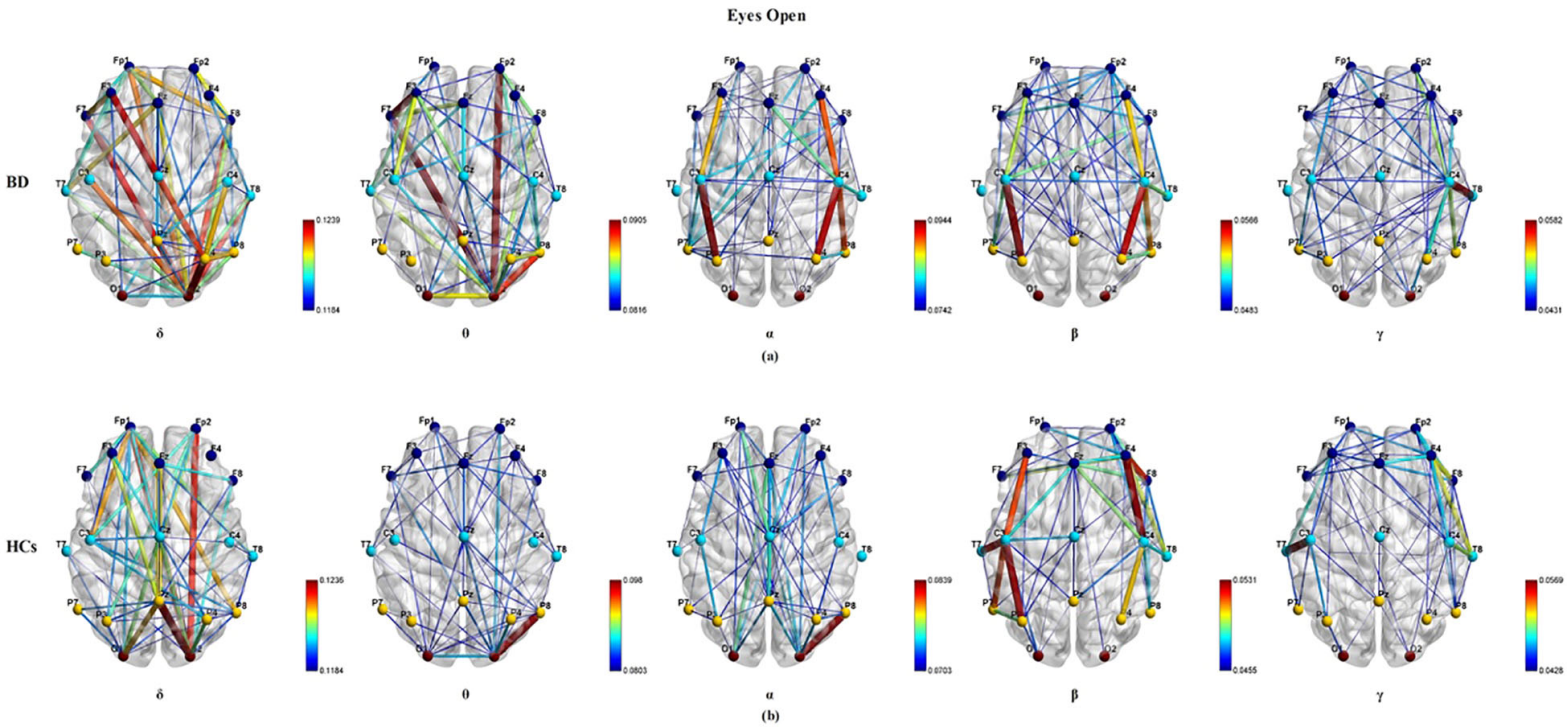
The visualization of the group average PLI functional connectivity for BD **(a)** and HCs **(b)** under the eyes open experimental paradigm across five frequency bands (δ, θ, α, β, and γ), with a sparsity of 0.3.

**Figure 2** illustrates the group average PLI functional connectivity patterns for BD patients (A) and HCs (B) under the eyes open paradigm condition. However, under the eyes open paradigm condition, when comparing the group average PLI across the δ, θ, α, β, and γ frequency bands, no significant differences were found between any pairs of electrode connections (P_FDR_ > 0.05).

**Figure 3:**
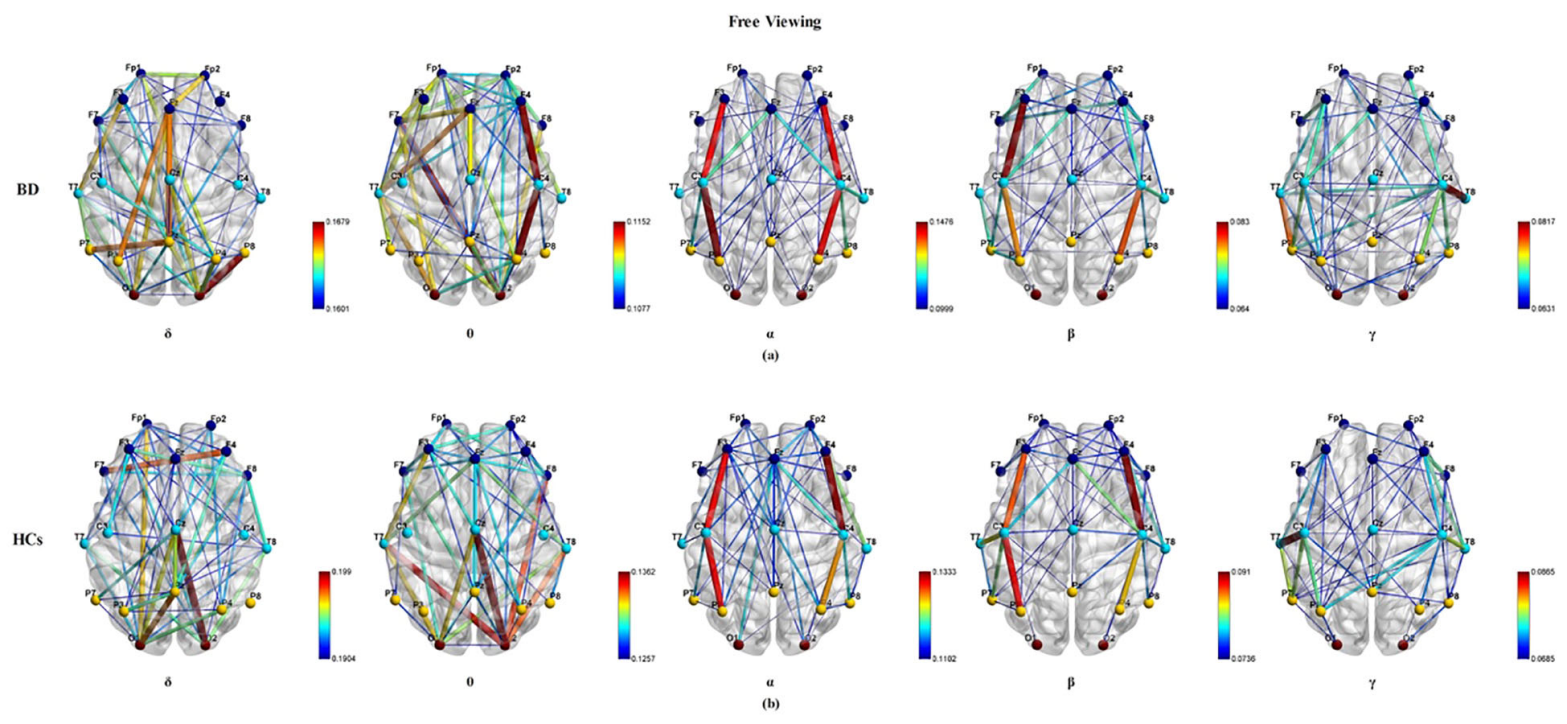
The visualization of the group average PLI functional connectivity for BD **(a)** and HCs **(b)** under the free viewing experimental paradigm across five frequency bands (δ, θ, α, β, and γ), with a sparsity of 0.3.

**Figure 3** illustrates the group average PLI functional connectivity patterns for BD patients (a) and HCs (b) under the free viewing paradigm condition. In δ frequency band, BD patients exhibited fewer and weaker long-range connections between the frontal cortical regions and parietal/occipital cortical regions than HCs (P_FDR<_ 0.05). In the θ frequency band, BD patients showed fewer and weaker long-range and short-range connections than HCs (P_FDR<_ 0.05). Functional connectivity between the two groups had no significant effects on the α, β, and γ frequency bands (P_FDR_ > 0.05). Within the three classical EEG experimental paradigms, the eyes closed paradigm exhibited significantly distinct PLI functional connectivity patterns across a broader range of frequency bands when comparing BD patients with HCs.

### 3.3 Significance analysis of features

Significant differences in the δ and β frequency bands were observed across all three classical experimental paradigms. Under the eyes closed paradigm, significant distinctions were primarily concentrated in the δ band (frontal, parietal, and occipital lobes), β band (parietal lobe), DE-δ (frontal, parietal, occipital, and temporal lobes), and DE-β (parietal lobe). In the eyes open paradigm, the features showing significant differences between BD patients and HCs were more dispersed across the δ, θ, α, β, and γ frequency bands. Within the free viewing paradigm, the significant distinctions were relatively scattered in the δ (frontal, parietal, and occipital lobes), θ (occipital lobe), α (occipital lobe), and β (parietal lobe) frequency bands. All three paradigms revealed notable differences in the δ band (frontal, parietal, and occipital lobes) between BD patients and HCs. However, the choice of paradigm influenced the observed outcomes in θ, α, β, γ, DE-θ, DE-α, DE-β, DE-γ, AAC, and dPAC features (**Table 2**).

**Table 2:** The electrodes significantly differ across the 12 features under the three experimental paradigms.

Paradigm	Brain Region	δ	θ	α	β	γ	DE-δ	DE-θ	DE-α	DE-β	DE-γ	AAC	dPAC
Eyes Closed	F	Fp1,Fp2,F3,F4,F7,F8,Fz					Fp1,Fp2,F3,F4,Fz,F7,F8						
	P	C3,C4,Cz,P3,P4,P7,P8,Pz			P3,P4,P8,Pz		C3,C4,Cz,P3,P4,P7,P8,Pz			P3,P4,Pz		Cz,Pz	P3
	O	O1,O2					O1,O2					O1,O2	
	T						T7,T8						
Eyes Open	F	F3,F7,Fz	F7	F7,F8			F3,F7,F8,Fz	F7	F7,F8	F3,F7	F7	Fz	
	P	C3,C4,Cz,P3,P4,Pz	C3		C3,Cz	C3,Cz	C3,C4,Cz,P3,Pz			Cz	Cz	P4,Pz	C4
	O	O1,O2	O1	O1,O2	O2		O1,O2		O1,O2	O2		O1	
	T						T7,T8						
Free Viewing	F	F3,Fz					F3,F7,Fz			F3		Fp1,F8	Fz
	P	C3,P3,Cz,Pz			P7		C3,C4,Cz,Pz					P8	
	O	O1,O2	O1,O2	O1,O2			O1,O2	O2	O1,O2	O2			
	T												

F, Frontal lobe; P, Parietal lobe; O, Occipital lobe; T, Temporal lobe; DE, Differential entropy; ACC, Amplitude-Amplitude Coupling; dPAC, Debiased Phase-Ampltiude Cross-Frequency Coupling.

### 3.4 Correlation with cognitive function

**Figure 4:**
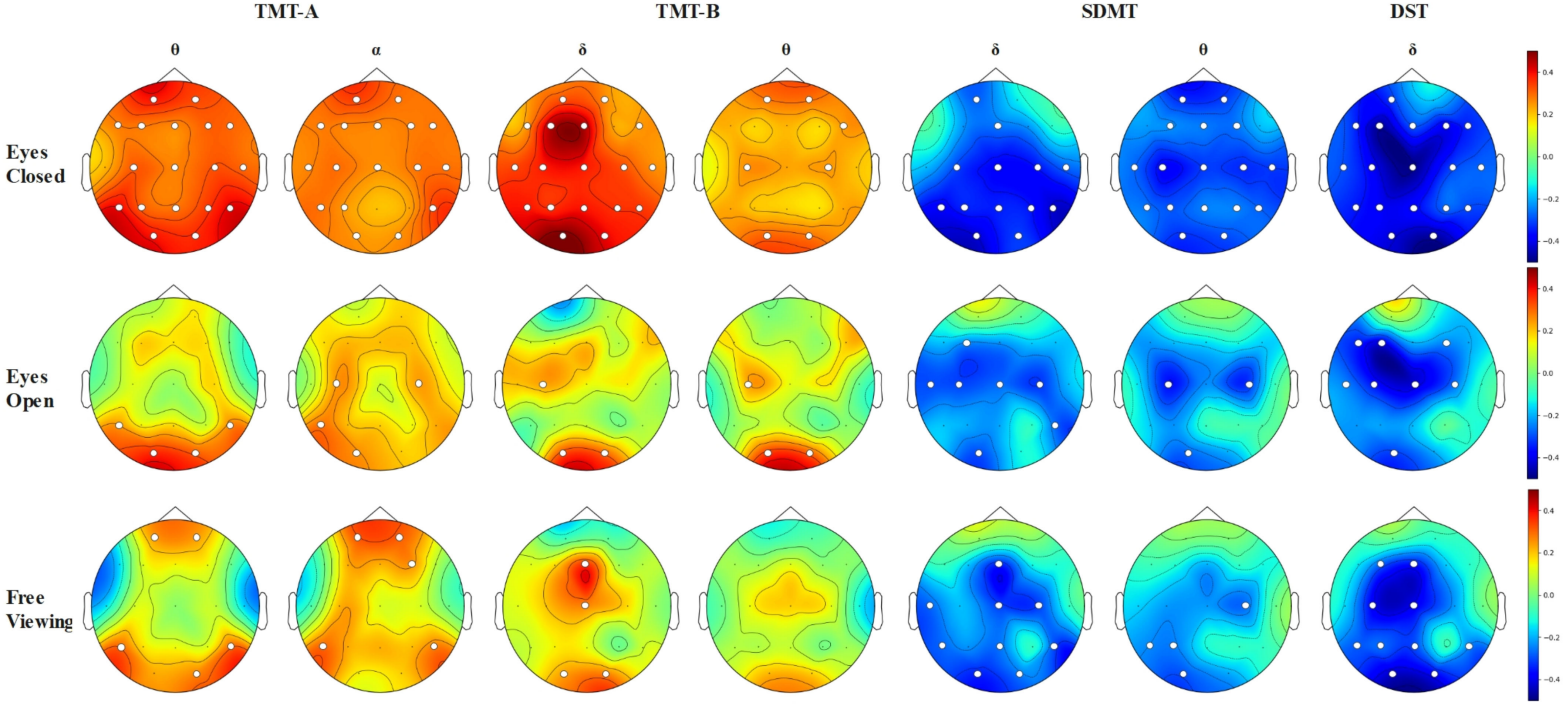
Topographical maps visualize the significant correlations between TMT-A, TMT-B, SDMT, and DST scores and features across different EEG frequency bands under various experimental paradigms. The white dots represent the electrode positions with significant differences.

TMT-A scores exhibited more positive correlations with θ and α PSD, DE-θ, and DE-α features across three experimental paradigms. TMT-B scores showed more positive correlations with δ and θ PSD and DE- δ, and DE-θ features across three experimental paradigms. SDMT scores displayed negative correlations with δ and θ PSD, DE-δ, and DE-θ features across three experimental paradigms. DST scores exhibited negative correlations with δ PSD and DE-δ features across three experimental paradigms (**Figure 4**).

We also observed differences in the number of significant features correlated with cognitive scores across the three experimental paradigms, with the highest number observed in the eyes closed paradigm and slightly fewer in the eyes open paradigm than in the free viewing paradigm. More brain regions showed significant correlations between EEG features and cognitive scores in the eyes closed paradigm compared to the eyes open or free viewing paradigms.

### 3.5 Classification

**Table 3** presents the results of BD recognition using six classifiers under three different experimental paradigms. Compared to the eyes open and free viewing paradigms, the eyes closed paradigm achieved the highest accuracy and F1 score in identifying BD. Under the eyes closed paradigm, the random forest classifier yielded the highest recognition accuracy of 79.43%, with F1 score of 76.82%. For the eyes open paradigm, the ensemble learning classifier demonstrated the best BD identification performance, achieving a recognition accuracy of 75.85% and F1 score of 71.28%. Similarly, the ensemble learning classifier achieved the best BD identification performance under the free viewing paradigm, with a recognition accuracy of 76.03% and F1 score of 72.6%.

**Table 3:** Accuracy and F1-score of different classifiers under different experimental paradigms.

Classifier	Metric	Experimental Paradigm		
		Eyes closed	Eyes Open	Free viewing
Ensemble	Acc(%)	77.26	75.85	76.03
	F1-score	74.46	71.28	72.56
KNN	Acc(%)	75.57	71.41	69.55
	F1-score	72.52	66.10	64.38
NaiveBayes	Acc(%)	70.63	67.79	65.58
	F1-score	69.82	63.34	58.80
Random Forest	Acc(%)	79.43	75.17	71.48
	F1-score	76.82	70.79	67.06
SVM	Acc(%)	77.78	75.58	75.60
	F1-score	74.87	70.39	71.16
DecisionTree	Acc(%)	68.89	65.35	61.62
	F1-score	66.18	61.10	58.31

KNN, K-nearest neighbours; SVM, Support vector machine; Acc, Accuracy.

## 4 Discussion

To our knowledge, this study represents the first attempt to recognize bipolar disorder by analyzing EEG signals using different experimental paradigms. We investigated the brain electrical activity of two groups of patients, BD and HCs subjects, under the conditions of eyes closed, eyes open, and free viewing experimental paradigms. Our research findings suggest that the eyes closed experimental paradigm may offer the highest discriminative power regarding brain functional connectivity analysis, cognitive function correlation analysis, and classification performance. There was no clear distinction between the free viewing and eyes open experimental paradigms regarding their superiority, which could be attributed to the specific free viewing paradigm adopted in our study.

First, in the eyes closed paradigm, the BD group exhibited more significant PLI frequency bands than the HCs group, indicating more pronounced differences in EEG signals under this condition. The BD group exhibited a higher group average PLI in the δ, θ, β, and γ frequency bands than the HCs under the eyes closed condition. A study has found increased coherence in the frontal and occipital cortices, particularly in the frontal regions, presenting more diffuse long-range brain connections than HCs (21), aligning with our findings. Another study revealed that patients with depression have enhanced PLI during sleep (59). This increased neural synchrony pattern may reflect network activity in a resting state. Excessive resting-state EEG activity has been described in bipolar affective disorder and may be related to higher depression levels (60). In the eyes open experimental paradigm, no significant differences were observed in the PLI across all frequency bands between the two groups. A study investigating the recovery of brain function impairments in young bipolar disorder patients during a stable emotional period revealed that under an eyes open experimental paradigm, these patients exhibited heightened activity across all frequencies (δ, θ, α, and β), suggesting deficits in visual-spatial processing (61). Similarly, another study aimed at distinguishing between female attention deficit hyperactivity disorder and female bipolar disorder patients found that the absolute θ power was higher in the bipolar disorder group than in the HCs group under an eyes open paradigm. However, no increase in absolute θ power was noted in the bipolar disorder group during a connect-the-dots task (62). These results differ from ours, potentially due to differences in study subjects and control groups, necessitating further research. There were fewer and weaker δ and θ connections than HCs under a free viewing paradigm. Previous studies using a stimulus-task paradigm have shown that at the onset of the stimulus, HCs had a higher PLI than bipolar disorder patients in the θ and α bands, with the PLI of bipolar disorder patients increasing later. However, connectivity in the β and γ bands only showed insignificant changes, indicating faster brain responses in HCs than in bipolar disorder patients (63). Another study using a self-referential memory (SRM) paradigm found that during self and other referential processing, HCs activated more brain connectivity regions than bipolar disorder patients and HCs had a higher PLI than bipolar disorder patients at the onset of the stimulus, suggesting that bipolar disorder patients ‘s self-cognition is impeded in the transmission and integration of interhemispheric information (48). Different free viewing paradigm contents might result in variations in functional connectivity across different frequencies. However, regardless of the paradigm, these consistently indicate bipolar disorder patients’ difficulties in attention, memory, and the integration of other brain resources. Our study revealed that the eyes closed paradigm could discern more functional connectivity differences between the two groups, whereas the free viewing experimental paradigm demonstrated fewer differences than those observed in the eyes closed paradigm between the groups across frequency bands.

Second, we conducted a significance analysis of the 12 extracted features and found that significant differences in the δ and β frequency bands were consistently observed across all three classical experimental paradigms. In the eyes-closed paradigm, notable differences were primarily observed in the δ band (frontal, parietal, and occipital lobes), β band (parietal lobe), DE-δ (frontal, parietal, occipital, and temporal lobes), and DE-β (parietal lobe). A study discovered that δ-β cross-frequency coupling may reflect the neural projection of the physiopathology of bipolar disorder; significant increases in dPAC and AAC were found in FP 2, with a linear correlation between dPAC in F3 and Mood Disorder Questionnaire scores were observed. Initially, P3’s dPAC was only related to Hamilton Depression Rating Scale scores (64). Our eyes closed experimental paradigm revealed significant differences in P3’s dPAC, consistent with these findings. Other studies also identified changes in the power of δ, β, and θ waves (65) Our analysis of 12 extensively used EEG features revealed that the selection of experimental paradigm affects the results of the significance analysis for observed features. The majority of EEG features (θ, α, β, γ, DE-θ, DE-α, DE-β, DE-γ, AAC, and dPAC) demonstrated variability in differences across the paradigms. And the brain regions exhibiting significant feature differences across different paradigms. Thus, the selection of a consistent and efficient experimental paradigm is crucial for researchers in exploring biomarkers for bipolar depression.

Third, this study found thatθ and α frequency bands positively correlated with scores on the TMT-A test.δ and θ frequency bands show positive correlations with TMT-B test scores and negative correlations with DST test scores.δ frequency band also negatively correlated with SDMT test scores. A study on the spontaneous α activity and visually evoked α responses in patients with bipolar disorder revealed a reduced spontaneous EEG α activity and a marked decrease in evoked α responses. These findings may be associated with the cognitive deficits characteristic of bipolar disorder (66). Prior research supports increased δ synchronization, such as heightened EEG δ activity in bipolar disorder than HCs, confirming the potential for poorer cognitive flexibility and executive function in patients with BD (67, 68). δ oscillations are crucial for cognitive functions related to focused attention, signal detection, recognition, and decision-making, and diminished δ responses may be a common feature of cognitive dysfunction in neuropsychiatric disorders (69) (70). Similarly, this study found that diminished δ responses were negatively correlated with the DST and SDMT scores. Another study indicated that reducing activity in the central θ band might help diminish cognitive control and maladaptive behavioral responses in patients with BD. The changes in the θ band observed in this study are consistent with these findings (C. M. 71).

Finally, we employed multiple widely used classical machine learning classifiers to assess the performance of three experimental paradigms in BD identification tasks. A review indicates that most studies on bipolar disorder have utilized classical machine learning models, such as SVM, Random Forest, and KNN, with 24 studies reporting accuracies ranging from 64% to 98% for machine learning models in BD identification tasks (72). The classifiers encompassed these mainstream machine learning models to ensure the reliability and comparability of our research findings. We found that all six classifiers achieved optimal classification performance under the eyes closed experimental paradigm, indicating the superiority of the eyes closed paradigm over the eyes open and free viewing paradigms (based on action observations) in BD recognition tasks. Moreover, the random forest classifier under the eyes closed paradigm exhibited the best classification performance, with a recognition accuracy of 79.43% and an F1 score of 76.82%.

## 5 Limitation

This study’s primary limitations were the small sample size and the fact that not all patients were medication-free. Another significant limitation is that our free viewing paradigm did not employ an emotional paradigm involving fear, anger, sadness, or other paradigms, which may have led to different results. Future research may explore the content of the free viewing paradigm in more detail.

## 6 Conclusion

Our study systematically analyzed the performance differences between the eyes closed, eyes open, and free viewing experimental paradigms in the BD identification task. We found that the eyes closed paradigm exhibited significant advantages over the eyes open and free viewing paradigms regarding feature significance, cognitive relevance, and classifier recognition accuracy. However, there was no significant differences between the eyes open and free viewing paradigms, possibly due to the use of the action observation-based free viewing paradigm. Other paradigms based on emotion induction and cognitive function deserve further exploration. Our findings suggest that the eyes closed paradigm, a simple and feasible experimental paradigm, holds greater clinical significance in EEG analysis for BD identification than the eyes open and action observation-based free viewing paradigms. Future research should investigate additional experimental paradigms designed based on emotional bias and cognitive function decline characteristics, providing a more diverse range of experimental paradigm references for BD identification studies.

## Data Availability

The original contributions presented in the study are included in the article/supplementary material. Further inquiries can be directed to the corresponding authors.
